# Structure of the Arteries

**Published:** 1843-06

**Authors:** 


*Structure of the Arteries.*—After a variety of conflicting and unsatisfactory accounts, Henle seems at length to have discerned such structures in the arteries as are adapted to the functions which experiment shows to be performed by them. His account of the general structure is briefly this:

1st, They have an epithelial lining, consisting of a very thin layer of elliptic or rhombic lameller cells, which are sometimes elongated into longitudinal spindle-shaped fibres.*

2d, There is, immediately external to this, a layer of peculiar tissue, the *striated* or *fenestrated* coat (corresponding to

---

* Remak and Reichert (Muller, Archiv. 1841, CLXXXVIII), hold, that these are not the innermost cells of the vessels, but that within these, and in actual contact with the blood, there is a layer of flattened, round, and polyhedral cells, with round, yellowish nuclei and nucleoli. On all these observations by Henle, see Reichert's remarks.

the *internal* coat of the older anatomists), consisting of a very thin, rather stiff, and brittle membrane, often perforated by numerous round or oval apertures, and bearing pale, flat, very narrow fibres, which have, for the most part, a longitudinal direction, and give it a peculiar delicately-striated appearance. This coat, which is often morbidly thickened, and when an artery is contracted, is commonly thrown into longitudinal folds, is produced by a metamorphosis of the epithelinm, whose cells, as their nuclei disappear, coalesce and form a homogeneous membrane, on which the fibres are afterwards deposited, and which at last, as the apertures in it enlarge, is completely removed, leaving the fibres free.

3d. In some arteries there is, next, a coat formed by a single layer of *longitudinal* granular *fibres*, flat and tolerably wide, analogous to a coat which is much more prominent in the veins.

4th. A coat composed of *circular fibres* (the *middle* or *elastic* coat of most foreign writers, the *muscular* coat of Hunter), which forms the chief part of the arterial wall, and comprises all that can be torn from it in a transverse direction. Its fibres are flat, clear, and granular, and break with abrupt ends. Each of them is commonly marked along its middle by dots scattered, or regularly arranged in a longitudinal row, or by a narrow streak: these are the remains of elongated nuclei, which have formed, as it were, the pattern, according to which the homogeneous membrane in which they lay has broken up into the flat fibres. The streaks formed of the elongated nuclei often branch and anastomose, so as to form that kind of net work which has led to this coat being mistaken for elastic tissue; whereas it is, in fact, the proper contractile coat of the artery, and is, in all respects of development and microscopic structure, similar to the layers of organic muscle in the stomach, &c.

5th. On its exterior there is a coat of genuine elastic tissue (*tissu jaune*, the *elastic coat* of Hunter): this exists, however, only in the larger arteries; and its thickness, in comparison with that of the preceding, diminishes in direct proportion to the size of the artery. The direction of its fibres varies greatly in different arteries.

6th, The *external cellular coat*, consisting of common cellular tissue, with longitudinal closely-woven filaments.—*Med. Ex.*, from *Lond. Med. Gaz.*

